# A simple approach to measure transmissibility and forecast incidence

**DOI:** 10.1016/j.epidem.2017.02.012

**Published:** 2018-03

**Authors:** Pierre Nouvellet, Anne Cori, Tini Garske, Isobel M. Blake, Ilaria Dorigatti, Wes Hinsley, Thibaut Jombart, Harriet L. Mills, Gemma Nedjati-Gilani, Maria D. Van Kerkhove, Christophe Fraser, Christl A. Donnelly, Neil M. Ferguson, Steven Riley

**Affiliations:** a MRC Centre for Outbreak Analysis and Modelling, Imperial College London, Faculty of Medicine, London, UK; b National Institute for Health Research Health Protection Research Unit in Modelling Methodology, Imperial College London, Faculty of Medicine, London, UK; c Center for Global Health, Institute Pasteur, Paris, France

**Keywords:** Forecasting, Rapid response, Branching process, Renewal equation, MCMC

## Abstract

Outbreaks of novel pathogens such as SARS, pandemic influenza and Ebola require substantial investments in reactive interventions, with consequent implementation plans sometimes revised on a weekly basis. Therefore, short-term forecasts of incidence are often of high priority. In light of the recent Ebola epidemic in West Africa, a forecasting exercise was convened by a network of infectious disease modellers. The challenge was to forecast unseen “future” simulated data for four different scenarios at five different time points. In a similar method to that used during the recent Ebola epidemic, we estimated current levels of transmissibility, over variable time-windows chosen in an ad hoc way. Current estimated transmissibility was then used to forecast near-future incidence. We performed well within the challenge and often produced accurate forecasts. A retrospective analysis showed that our subjective method for deciding on the window of time with which to estimate transmissibility often resulted in the optimal choice. However, when near-future trends deviated substantially from exponential patterns, the accuracy of our forecasts was reduced. This exercise highlights the urgent need for infectious disease modellers to develop more robust descriptions of processes – other than the widespread depletion of susceptible individuals – that produce non-exponential patterns of incidence.

## Highlights

• Our simple approach relies on very few parameters and minimal assumptions
• Subjective choice of best training period improved forecasts
• Despites its simplicity, our model forecasted well under a range scenarios.
• This approach can be a natural 'null model' for comparison with methods.

## 1 Introduction

In epidemiology, and particularly in the context of outbreaks, mathematical modelling is now frequently used to forecast future incidence (Chretien et al., 2015, Nsoesie et al., 2014). Such forecasts were initially performed to improve the situational awareness of key stakeholders. Increasingly, forecasting incidence is used in the context of advocacy planning, to monitor the situation, and to help implement, prioritise and evaluate control strategies. During the recent Ebola epidemic in West Africa, such forecasts were almost continuously performed: many were shared with policy makers with some published in peer-reviewed literature (WHO Ebola Response Team, 2015a, Meltzer et al., 2014, WHO Ebola Response Team, 2014, Gomes et al., 2014, Merler et al., 2015).

While all methods for forecasting future incidence seek to characterise the central predicted trend and the dispersion around it based on covariates, they vary according to the nature of the underlying model, with some methods relying on a purely statistical approaches (Goldstein et al., 2011) and some relying on a mechanistic models of disease transmission (Meltzer et al., 2014). Recent forecasting exercises in the context of influenza (Influenza Forecasting, 2017), Dengue (Dengue Forecasting, 2017) or Chikungunya (Chikungunya Forecasting, 2017) highlight the diversity of possible models with some clearly belonging to one of the aforementioned categories while others take a more nuanced approach perhaps best described as semi-mechanistic. In all models, a careful balance must be reached between obtaining accurate forecasts while accounting for all uncertainties, both in the data themselves and in the dynamics of transmission.

During the recent Ebola epidemic, our team helped support the World Health Organization (WHO) and the Ministries of Health of the three most affected countries (Guinea, Liberia and Sierra Leone). In a wide collaborative effort, we were able to gain valuable insights into the transmissibility, epidemiology and impact of intervention strategies (WHO Ebola Response Team, 2015a, WHO Ebola Response Team, 2014, WHO Ebola Response Team, 2015b, Nouvellet et al., 2015, WHO Ebola Response Team, 2016, Garske et al., 2017). We were also involved in producing regular forecast of future incidence (e.g. WHO Ebola Response Team, 2015a, WHO Ebola Response Team, 2014), using a semi-mechanistic model based on a renewal equation (Fraser, 2007).

As the Ebola epidemic was declining, the Research and Policy for Infectious Disease Dynamics (RAPIDD) program, from the US National Institute of Health’s Fogarty International Center, gave eight teams (including us) the opportunity to assess their models against simulated data. Simulated data (based on Gomes et al., 2014, Merler et al., 2015) for 4 outbreak scenarios, differing in the assumptions underlying transmissibility and degree/quality of data reporting, at 5 different time-points during the outbreak were provided together with ‘field reports’ outlining the epidemiological situation (see SI.1). For each scenario and time-point, we were tasked with providing short-term forecasts (4 weeks into the future) and an estimate of the current level of transmissibility. Here we present the method used by the ‘Imperial College Team’ and how it performed.

## 2 Methods

At each of the five time points, and for each scenario, we were provided with a case-count dataset that consisted of weekly counts of newly confirmed cases (Table 1). A field report was also provided, containing information on interventions, e.g.: timing of a recently implemented intervention or increased bed capacity (see SI.1).

**Table 1:** Estimated instantaneous reproduction numbers (*R*_*t*_) and serial intervals (in days) for the 5 time-points and 4 scenarios.

Scenario	Line-list	Case-count	Field-report	Time-point	R0 (median)	R0 (IQR)	SI (median)	SI (IQR)
1	✓	✓	✓	1	1.03	[0.86; 1.25]	15.4	[11.3; 18.7]
				2	1.33	[1.27; 1.40]	13.3	[10.1; 16.0]
				3	0.87	[0.85; 0.90]	12.5	[9.8; 14.8]
				4	0.87	[0.85; 0.90]	12.5	[9.8; 14.8]
				5	0.79	[0.75; 0.82]	12.7	[10.3; 14.7]

2		✓	✓	1	1.62	[1.49; 1.75]	14.2^a^	
				2	0.89	[0.86; 0.92]		
				3	1.00	[0.96; 1.05]		
				4	0.91	[0.89; 0.94]		
				5	0.72	[0.70; 0.74]		

3		✓	✓	1	1.69	[1.55; 1.83]	14.2^a^	
				2	1.28	[1.20; 1.37]		
				3	1.32	[1.28; 1.37]		
				4	1.05	[1.02; 1.08]		
				5	0.69	[0.67; 0.71]		

4		✓	✓	1	1.43	[1.29; 1.58]	14.2^a^	
				2	1.39	[1.31; 1.46]		
				3	1.12	[1.09; 1.15]		
				4	0.88	[0.85; 0.91]		
				5	0.98	[0.96; 0.99]		

IQR: interquartile range.

a Indicated that in the absence a line-list, the distribution of the serial interval was taken from WHO Ebola Response Team (2015a). Unknown at the time of challenge, accuracy of data and reports progressively decreased from scenario 1 to scenario 4.

**Fig. 1:**
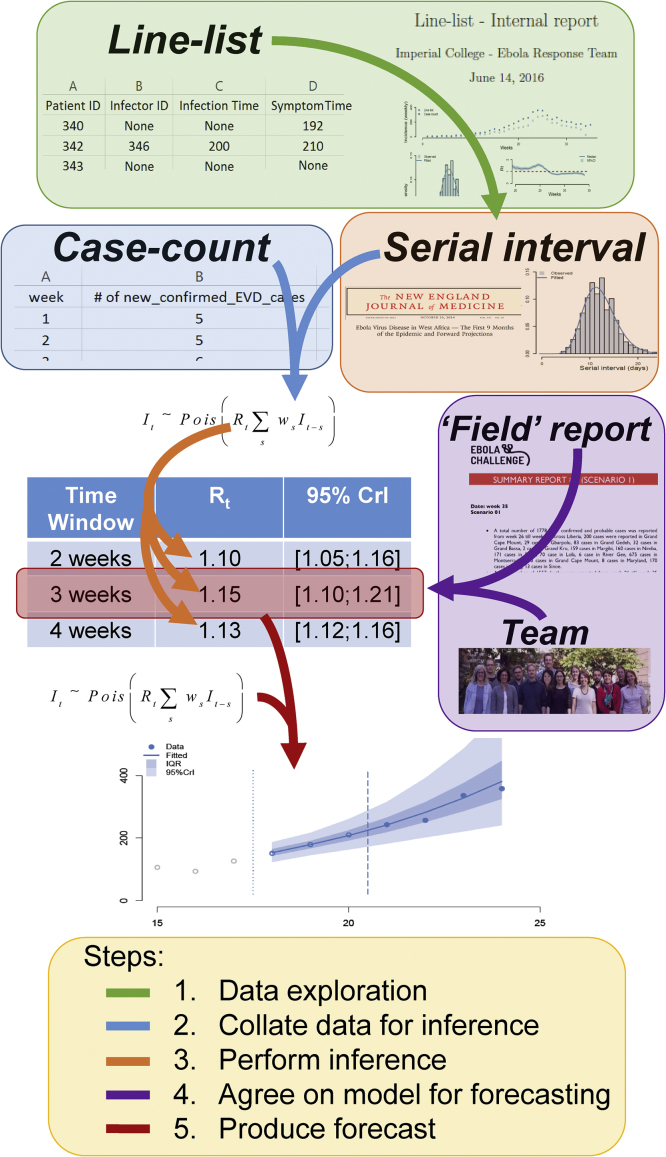
Schematic of our forecasting process. First the line-list, if present, was used to 1) estimate the serial interval distribution, and 2) gain insight into the drivers of transmission and give us better situational awareness. Then we used the incidence of confirmed cases provided in the case-count and the serial interval distribution (either from the literature or from the line-list) to estimate the instantaneous reproduction number *R*_*t*_. The estimation relied on the renewal equation and assumed transmissibility to be constant during a chosen time-window (either 2, 3 or 4 weeks). Then based on the ‘field report’ provided, assessment of the line-list (when present), and general trends in past incidence, an *R*_*t*_ estimate was chosen (by choosing a time-window) to be used to predict 4 weeks of future incidence. The same renewal equation was used for forecasting relying on posterior distribution of the estimated *R*_*t*_.

**Fig. 2:**
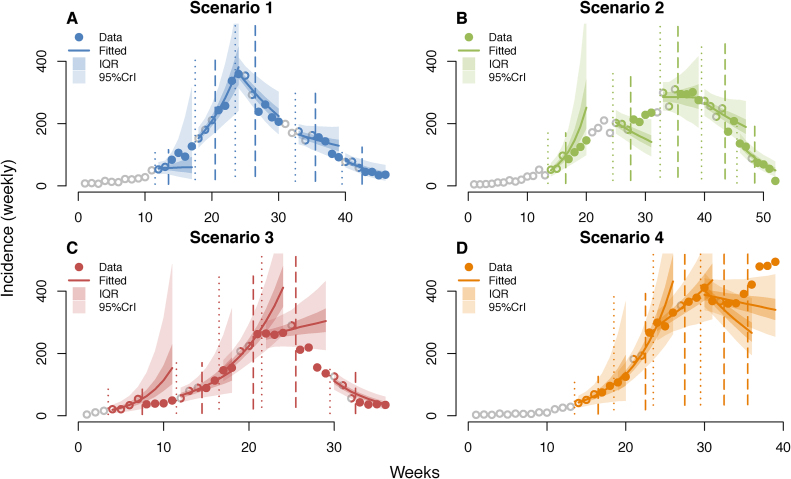
Weekly incidence of confirmed cases for each scenario with forecasts (A-D). Dots represent the observed incidence while the solid lines show the median prediction (shaded envelopes show the interquartile range, IQR, and the 95% credible interval, CrI) at each time-point. Coloured open dots show the observed incidence used for inferring the reproduction numbers between the start (vertical dotted lines) and the end (dashed vertical lines) of the chosen time-windows. Filled coloured dots show the observed incidence in the forecast periods. Weekly observations predicted and subsequently used for inference are shown as solid dots (e.g. in scenario 3, the incidence predicted for the 3rd time-point overlap with incidence used for the 4th time-point forecasts). Grey open dots were not used for inference and never predicted.

Our approach was to estimate the current reproduction number (the average number of secondary cases generated by a typical infected individual, *R*_*t*_) and to use that to forecast future incidence (Fig. 1, Fig. 2). The current reproduction number was estimated using the case-count dataset, assuming constant transmissibility during a chosen time-window (see the Estimation and Forecast sections below).

**Fig. 3:**
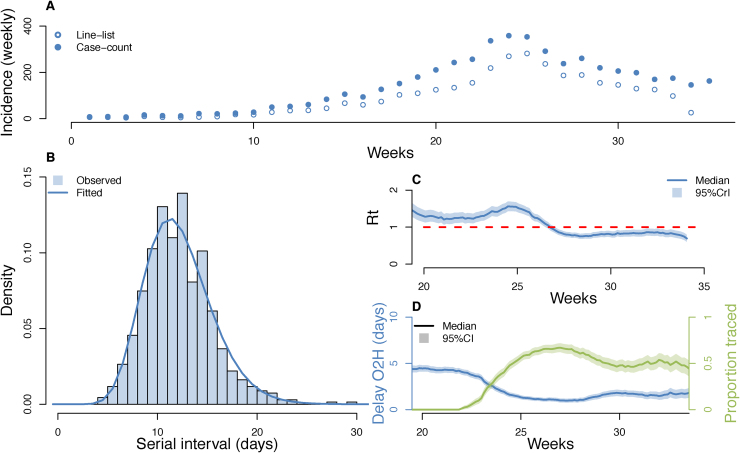
Sample of information extracted from the line-list to inform our analysis. The example shown refers to the fourth time-point of scenario 1. A. Weekly incidence of confirmed cases from the line-list and the case-count data. B. Serial interval distribution observed and fitted using line-list data. C. Daily estimates of the reproduction number (*R*_*t*_) (median and 95% CrI) on two-week sliding time-windows. The red horizontal dashed line represents the threshold 1, below which an epidemic is considered under control. D. Median (solid line) delay from onset to hospitalisation (blue curve associated with left y-axis) and proportion of cases in the line-list who were under surveillance prior to infection due to contact tracing activities. The shaded areas show the 95% confidence intervals (CIs). (For interpretation of the references to colour in this figure legend, the reader is referred to the web version of this article.)

For scenario 1, we were also provided with a line-list. The line-list contained detailed data for each individual and was used exclusively to infer a serial interval distribution and gain epidemiological insights into the current situation (see preliminary analyses and Fig. 3). The line-list focused on confirmed cases, and was affected by both under-reporting and delays in reporting (see ‘Preliminary analyses’ below).

### 2.1 Estimation of the reproduction number

The reproduction number used to forecast future incidence was estimated from the case-count data.

Several methods to estimate the reproduction number exist, e.g. see Van Kerkhove et al. (2015) for various methods linked to the estimation of the basic and effective reproduction of Ebola virus. Here we relied on a well-established and simple method that assumed the daily incidence, *I*_*t*_, could be approximated with a Poisson process following the renewal equation (Fraser, 2007):

$$I_{t}∼Pois\left(R_{t}\sum_{s=0}^{t}I_{t-s}\omega_{s}\right),$$

where *R*_*t*_ is the instantaneous reproduction number and *ω* the serial interval distribution. From this a likelihood of the data given a set of model parameters can be calculated, as well the posterior distribution of *R*_*t*_ given previous observations of incidence and knowledge of the serial interval (Cori et al., 2013). The serial interval was assumed to be gamma distributed with parameters taken either from the literature (WHO Ebola Response Team, 2015a) (i.e. for scenario 2–4), or estimated from the line-list (i.e. scenario 1, see preliminary analyses below).

We used this approach to estimate *R*_*t*_ over three alternative time-windows defined by assuming a constant *R*_*t*_ for either the 2, 3 or 4 weeks prior to the most recent data-point. We made no assumptions regarding the epidemiological situation and transmissibility prior to each time-window. Therefore, no data prior to the time-window were used to estimate *R*_*t*_ and instead we jointly estimated *R*_*t*_ as well as back-calculated the incidence before the time-window. Specifically, we jointly estimated the *R*_*t*_ and the incidence level 100 days before the time-widow. Past incidence was then calculated using the known relationship between the serial interval, growth rate and reproduction number (Wallinga and Lipsitch, 2007). The joint posterior distribution of *R*_*t*_ and the early epidemic curve (from which forecasts were generated) was inferred using Markov Chain Monte Carlo (MCMC) sampling.

Because the case-count data reported cases by week rather than by day, we inferred the daily incidence data using data augmentation (Cauchemez et al., 2006, O’Neill and Roberts, 1999). We assumed that cases followed a multinomial distribution within the week. At each iteration of the MCMC, a new distribution of cases within each week was proposed by sequentially shifting a third of cases by ±1 day and the likelihood reassessed to accept/reject the proposed distribution of cases within the week.

### 2.2 Forecast for future incidence

We simulated future incidence using a branching process model based on the renewal equation (Fraser, 2007), assuming the same Poisson offspring distribution used for inference (see equation above). The serial interval for forecast was identical to that used in inferring the reproduction number above. For each simulation, the initial incidence and *R*_*t*_ were sampled from the joint posterior distributions (see section “Estimation of the reproduction number”). Medians, 95% credible intervals (95% CrIs) and interquartile ranges (IQRs) were generated from 10,000 simulations.

Estimated *R*_*t*_ and incidence forecasts for the 4 weeks ahead were produced for each of the three time-windows, one of which was chosen for ‘submission’ to the model assessment exercise. The choice of time-window was somewhat subjective, based on the field report provided, an assessment of the line-list (see below, we did not use specific numerical output from line-list analysis), and an assessment of the general trends in incidence. The choice of the time-window ultimately aimed to balance more precise estimation (favouring long windows with more data), with accurately reflecting the most recent and anticipated future transmissibility (favouring short windows limited to very recent data).

### 2.3 Preliminary analyses

Preliminary analyses relied on the line-list, and mirrored to some extent the work-flow we used in 2014-15 to analyse the West African epidemic. For this exercise, the line-list included time-stamped clinical events for each patient from exposure until death or recovery: reported exposure, symptom onset, hospitalisation, entry in an Ebola Treatment Unit; whether the patient was under surveillance from contact tracing activities prior to infection and how many of the patient’s contacts were being followed (reflecting forward contact tracing); who was the likely infector; whether the patient died, and if so, whether the burial was conducted safely.

First, incidence time-series were computed and plotted. However, the line-list was unreliable to assess incidence (Fig. 3A) due to under-reporting throughout the epidemic (e.g. case-count dataset shows much higher incidence than the weekly aggregated counts from the line-list). Additionally and especially relevant for real-time forecasting, the most recent few weeks of data in the line-list were even less reliable; this simulated the delays in updating the line-list. Therefore, while we analysed the line-list to gain epidemiological insights, the line-list was not directly used to assess the transmissibility used in the forecast.

Then, using pairs of infector-infectees for which dates of symptom onset were known, we computed the observed serial interval (the time between symptom onset of a case and symptom onset of their infector). We fitted a gamma distribution to these observed serial intervals by maximum likelihood (WHO Ebola Response Team, 2014). Using this serial interval distribution, the instantaneous reproduction number (*R*_*t*_) was estimated on a daily basis using the EpiEstim R package (Cori et al., 2013) with a 2-week sliding window. This reproduction number provided insights into the trend in transmissibility, but was not used for forecasting.

Additionally, for each patient, we extracted all delays using the clinical histories provided. To estimate temporal variation in the delays, we calculated for each day the median delay over the previous 2 weeks. Then, for each delay, we plotted the daily delay (median) against the daily *R*_*t*_ above (median). The correlations between *R*_*t*_ and the various delays were examined visually (and with univariable linear models) to identify possible drivers of changes in transmission.

We also quantified the number of times a patient was recorded as being an infector in the line-list. The incomplete nature of the data available precluded direct estimation of the reproduction number from such contact data, but it still allowed us to compare transmission between different groups. For instance, we compared our transmission rates from patients who had safe burials versus patients with unsafe burials (assuming data were missing at random).

The line-list preliminary analyses retrospectively informed us on the impact of interventions or situational changes (e.g. observed increase in safe burials), which were not reflected in the ‘field report’. For instance, Fig. 3C–D helped us build a narrative where contact tracing activities appear to decrease the delay between onset of symptom and hospitalisation, resulting in decreased transmissibility, presumably from quicker case isolation. Therefore, any future increase in contact tracing activities or evident recent decreasing trend in the delay from symptom onset to hospitalisation would lead us to expect a further decrease in transmissibility. More generally, if the daily instantaneous reproduction number estimated from the line-list showed a consistently decreasing trend (as opposed to a stable trend), again we would expect a further decrease in transmissibility (as opposed to relatively constant trend in transmissibility): essentially we conducted a subjective assessment of accelerations/decelerations in transmissibility.

### 2.4 Retrospective analyses

After the challenge ended, we were able to retrospectively assess the quality of our forecasts. For each scenario and each time-point, we extracted the number of observed weekly incidence counts that fell in our predicted IQR. IQR was chosen as it provides a good measure for accuracy in the central trend and formed the basis for the performance metrics used to compare model performances in this challenge. For a model which forecasts well, we expect 50% of our predicted incidence to fall within the IQR. The 95% CrIs are presented in figures to assess the estimation of uncertainties around our forecasts.

For each forecasting time-point, we measured the fit of forecasts versus observations using the mean squared error (MSE). This allowed us to define the optimal time-window (i.e. 2, 3 or 4 weeks) for our model, which we compared to our subjective choice of time-window (for each scenario/time-point). We were therefore able to assess the appropriateness of both the model and – to some degree – our choice of time-window.

The measure of fit, corrected for the number of points (n) used for fitting (i.e. MSE×(n)/(n−1), equivalent to the unbiased sample variance), was also calculated within the time-windows, quantifying the fit of the model to the data used in the inference of *R*_*t*_. Therefore, we could define the optimal time-windows in terms of fit used for the inference of *R*_*t*_. In this *post hoc* report, we call this time-window the ‘*naïve rational*’ time-window: *‘rational’* as it is entirely and solely determined by the case incidence, however *‘naïve’* as it does not account for any other source of information which may be available. For instance, the ‘*naïve rational*’ approach would overlook valuable information such as knowing that contact tracing causes lower onward transmission, and that contact tracing efforts have recently increased.

## 3 Results

### 3.1 The 4 scenarios

In retrospect, scenario 1 was perhaps the most straightforward of the 4 scenarios from a forecasting perspective (Fig. 2). It appeared to be simulated from a prolonged and consistent exponential growth phase, followed by a sharp drop in transmissibility and then a consistent exponential decay phase. The other 3 scenarios were more complex, consisting of multiple phases with clearly differing rates of transmission and, in retrospect, little evidence of sustained exponential growth (or decline).

### 3.2 Forecasting scenario 1

In terms of forecast, with the exception of the first time-point, our method performed well (Fig. 2A). Overall, 11 observed incidence points (out of 20) were included in our predicted IQR (well within the expected range). All observed incidence points were included in our predicted 95% CrI. In retrospect, if we had been able to choose the correct time-windows for every forecasts, up to 16 out of the 20 points could have been included in our predicted IQR. Based on our simple measure of fit, our choice of time-window was optimal for 3 time-points (out of 5) and outperformed the *naïve rational* choice for 3 time-points (see Fig. S1-2 in SI.2).

The comparison of the line-list data to the case-count data showed that not all confirmed cases entered the line-list (Fig. 3A), and these discrepancies were especially important during the most recent few weeks included in the datasets. We therefore concluded that the case-count data were more reliable than the line-list data for forecasting. However, the line-list data were key in estimating the serial interval at each time-point (Fig. 3B and Table 1), with our best estimate (i.e. based on the most complete dataset) of the median as 12.7 days (95% CrI [10.3; 14.7]). Additionally, the line-list data allowed us to better understand the drivers of transmissibility. For instance, increased contact tracing was highly correlated with a reduction in the delay from onset to hospitalisation (Fig. 3D). Furthermore, cases entering the line-list through contact tracing had a significant lower case reproduction number (result not shown). This allowed us to hypothesise that contact tracing allowed quicker identification of cases, resulting in quicker hospitalisation and isolation. In turn this was ultimately reflected in lower intensity of transmission (*R*_*t*_) at the population level.

However, subjective choices driven by analysis of the line-list did not always improve forecasts. An increase in the proportion of safe burials recorded in the line-list close to the first time-point drove us to mistakenly choose a short time-window for our first forecast.

### 3.3 Forecasting scenario 2

The forecasts for scenario 2 were less accurate than for scenario 1, with good performance in the three most recent time-points of the outbreak (Fig. 2B) but significant inaccuracies in the first two. Out of 20 observations, 8 were included in our predicted IQR; and 15 observed incidence points were included in our predicted 95% CrI. The former only improved to 9 points when choosing the best time-window. Based on our measure of fit, we chose the optimal time-windows for 3 time-points and our choice was always better than the *naïve rational* choice (see Fig. S1-2 in SI.2).

### 3.4 Forecasting scenario 3

The forecasts were comparable in performance to that of scenario 2 (Fig. 2B–C), with inaccurate predictions for 2 time-points during the initial growth as well as the down-turn of the outbreak. Other time-points were well predicted. Only 6 out of 20 observations were included in our predicted IQR, due to the poor fit during the first and third time-points. 17 observed incidence points were included in our predicted 95% CrI. However, even in retrospect, those inaccurately forecasted time-points would be extremely difficult to predict due to sharp changes in incidence. With an optimal choice of time-window, 9 observations would have been included in the predicted IQR. We only chose the optimal time-window in 2 instances, but we still outperformed the *naïve rational* choice 3 times (see Fig. S1-2 in SI.2).

### 3.5 Forecasting scenario 4

While scenario 4 forecasts were good for the first 3 time-points, the most recent 2 time-points were poorly predicted. Overall, 9 observations were included in our predicted IQR; with 15 observed incidence points included in our predicted 95% CrI. By choosing an optimal time-window, up to 16 observations would have been in the predicted IQR. We chose the optimal time-window only once, while the *naïve rational* choice was optimal at 3 time points, associated with 15 observations included in the predicted IQR (see Fig. S1-2 in SI.2).

## 4 Discussion

We have described our participation in a blinded forecasting exercise based on the recent West African Ebola epidemic, predicting in real-time future weekly case incidence. The simulated weekly incidence often fell within the IQR of our forecast (i.e. overall out of 80 data-points, 34 were included in our IQRs), and within the 95% CrI (i.e. overall out of 80 data-points, 67 were included in our 95% CrIs). Therefore our simple approach performed well with good accuracy in forecasting the central tendency in the data, as well as characterising accurately the uncertainty. Our approach worked best when near-future patterns of incidence were well described by an exponential trend. Our results highlight the need for a better understanding of infectious disease transmission processes that lead to non-exponential changes in incidence. As highlighted by Viboud et al., (2016), such non-exponential growth may reflect heterogeneity in contact rate, early onset of interventions or changes in population behaviour.

As illustrated here, forecasting can be challenging as we intend to produce accurate trends while correctly accounting for uncertainties surrounding both the data and the transmission dynamics of the disease. As we did during the recent West African outbreak, we chose a simple approach, which offers the benefit of robustness at the cost of a weakly mechanistic underlying model. Therefore, although our model could in theory be used to measure the impact of control strategies (see International Ebola Response Team, 2016), it would not be the optimal tool to achieve this objective.

While simple, robust and performing well, our method required us to subjectively choose a time-window. This choice allowed us to account for additional information from the ‘field report’ provided, the line-list (when available), and an assessment of the general trends in recent incidence. We showed that this procedure was in most instances superior to a method relying on the fit of data within the time-window (i.e. ‘*naïve rational*’ strategy). While an alternative to this subjective method would be to use a more complex model (i.e. a more detailed description of transmission mechanisms), the benefit of simplicity was viewed as favourable compared to perhaps more robust but highly uncertain and more complex modelling.

Our analyses showed that the ‘field report’, the provided line-list and/or our current understanding of the disease could be misleading. For instance, we initially assumed that safe burial practices would considerably limit onward transmission. For the first time-point for scenario 1, the line-list revealed a recent increase in safe burial practices, leading us to mistakenly choose a time-window associated with lower estimated transmission intensity (Fig. 1A). This error reflected the limits of our subjective understanding of disease transmission.

While not always optimal in retrospect, the human component of our methodology needs to be further acknowledged in the process of forecasting, at least to assess its impact. While trying to minimise it, ultimately, the choice of a model and its assumptions implies a subjective understanding of the disease dynamics. For instance weather forecasting, which has seen a sustained increase in forecast accuracy in the past 30 years (Haiden et al., 2015), typically operates with a combination of models (ensemble forecasting) but still acknowledges a human component, which is progressively diminished in importance (Roebber and Bosart, 1996). Given that disease dynamics are ultimately greatly influenced by human behaviour, it seems in fact quite natural to have a ‘human’ component in infectious disease modelling. ultimately, we think that the field should strive to accurately report the subjective choices within the methodology for the sake of both simplicity and reproducibility and rely on ensemble forecasts (rather than forecasts from a single model). We also think it is crucial that researchers are upfront about the subjective nature of their forecasts.

We enjoyed participating in the challenge and we see considerable value is similar future exercises as a way of maintaining scientific focus in this challenging topic between outbreaks. Many of the issues faced during this exercise were similar (although to a lesser extent) to those experienced during the recent West African outbreaks (see Cori et al., 2017, for a full discussion of the challenges of data analyses in real-time during outbreaks). To name a few: data cleaning remains a significant task (as errors were added to the simulated data) and the choice of using a data-rich line-list versus a more limited case-count dataset (which are not necessarily consistent with one another) for forecasting is challenging (see WHO Ebola Response Team, 2015a, WHO Ebola Response Team, 2014 for instance). Also, the quick pace of analyses and required updates could easily result in human and communication errors.

To conclude, the forecasting challenge proved useful to further reflect on the methodology we had used during the recent West African Ebola epidemic, a luxury of time sometimes difficult to achieve in the midst of an emergency. Furthermore, the joint work on this project (e.g. workshops organised by RAPIDD) has allowed us to explore new ideas as well as further our links with external partners who could prove crucial (in terms of preparedness) should another outbreak requiring rapid forecasting occur.

## Conflict of interest

‘None declared’.
