# Supplementary material for: A simple approach to measure transmissibility and forecast incidence
Source: Epidemics. 2018 Mar;22:29–35. doi: 10.1016/j.epidem.2017.02.012 (PMC5871640; doi:10.1016/j.epidem.2017.02.012)
Supplement: Supplementary file 1 [file mmc1.pdf]

EBOLA CHALLENGE

SUMMARY REPORT #1 (SCENARIO 1)

Date: week 13
Scenario 01

- The first confirmed cases of Ebola virus disease(EVD) was reported on day 1 in Gbarpolu.
- A total number of 324 EVD confirmed and probable cases was reported till week 13 across Liberia, 203(the majority) of the cases were reported in Grand Cape Mount, 28 cases in Gbarpolu, 1 case in Margibi, 79 cases in Bomi, 1 case in Lofa, 3 cases in Montserrado, 9 cases in Bong. So far the reported cases are only found in the counties in the northwest of Liberia.
- A total number of 178 deaths were reported till week 13 across Liberia, among which 116 were in Grand Gedeh, 16 in Gbarpolu, 43 in Bomi, 3 in Bong.
- A total number of 13 health care workers(HCW) confirmed and probable cases were reported, 11 in Grand Cape Mount, 2 in Bong. 6 of them were among the fatalities count up to week 13.
- Traditional burial practices were observed among all communities which involves washing/touching/kissing the bodies during ceremony. The practices are confirmed to contribute to the spreading of EVD. No effective safe burial protocol has been enforced till week 13.
- No contact tracing cases were followed till week 13
- No Ebola Treatment Unit(ETU) were opened till week 13. No plans of opening ETUs in near future.
