# Supplementary material for: A simple approach to measure transmissibility and forecast incidence
Source: Epidemics. 2018 Mar;22:29–35. doi: 10.1016/j.epidem.2017.02.012 (PMC5871640; doi:10.1016/j.epidem.2017.02.012)
Supplement: Supplementary file 2 [file mmc2.pdf]

Supplementary Information 2

A simple approach to measure transmissibility and forecast incidence

Pierre Nouvellet[1,2], Anne Cori[1], Tini Garske[1], Isobel M Blake[1], Ilaria Dorigatti[1], Wes Hinsley[1], Thibaut Jombart[1,2], Harriet L Mills[1], Gemma Nedjati-Gilani[1], Maria D Van Kerkhove[1,3], Christophe Fraser[1,2], Christl A Donnelly[1,2], Neil M Ferguson[1,2], Steven Riley[1,2]

1: MRC Centre for Outbreak Analysis and Modelling, Imperial College London, Faculty of Medicine, London, UK.
2: National Institute for Health Research Health Protection Research Unit in Modelling Methodology, Imperial College London, Faculty of Medicine, London, UK.
3: Center for Global Health, Institute Pasteur, Paris, France

Below we present figures analogous to Figure 2 in the main text, but relying on either the optimal time-windows or the naïve rational choice of time-windows.

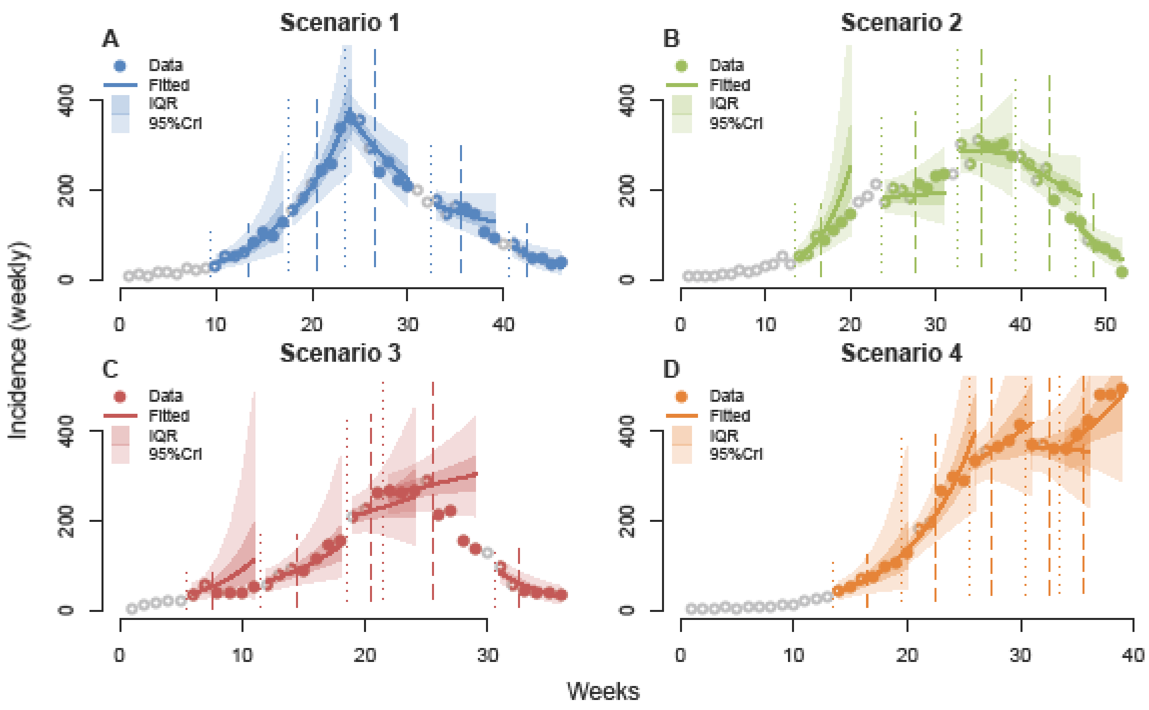

Figure SI.2.1: As in Figure 2, but for the optimal time-windows.

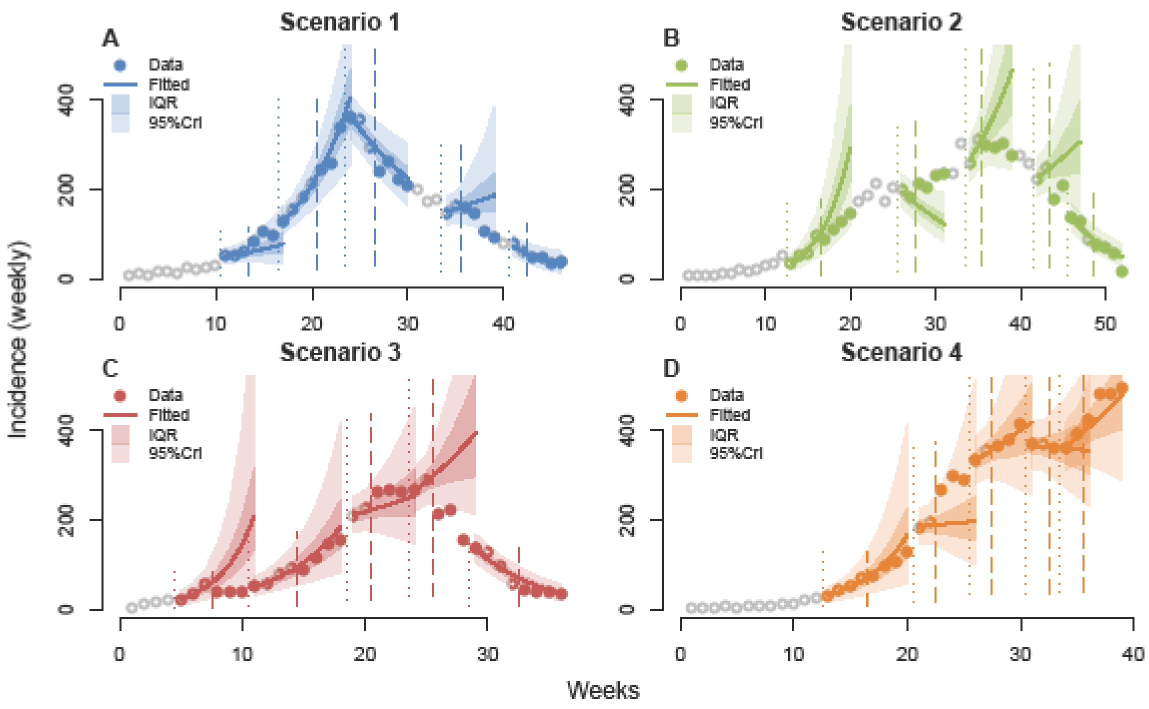

Figure SI.2.2: As in Figure 2, but for the naïve rational choice of time-windows.
